# Dietary patterns, gender, and weight status among middle-aged and older adults in Taiwan: a cross-sectional study

**DOI:** 10.1186/s12877-017-0664-4

**Published:** 2017-11-21

**Authors:** Miriam Adoyo Muga, Patrick Opiyo Owili, Chien-Yeh Hsu, Hsiao-Hsien Rau, Jane C-J Chao

**Affiliations:** 1 School of Nutrition and Health Sciences, College of Nutrition, Taipei Medical University, Taipei, Taiwan; 2 Department of Nutrition and Dietetics, University of Eastern Africa, Baraton, Eldoret, Kenya; 3 Department of Public Health, School of Health Sciences, University of Eastern Africa, Baraton, Eldoret, Kenya; 4 Institute of Environmental and Occupational Health Sciences, School of Medicine, National Yang-Ming University, Taipei, Taiwan; 5 Department of Information Management, National Taipei University of Nursing and Health Sciences, Taipei, Taiwan; 6 Master Program in Global Health and Development, College of Public Health, Taipei Medical University, Taipei, Taiwan; 7 Graduate Institute of Biomedical Informatics, College of Medical Technology, Taipei Medical University, Taipei, Taiwan; 8 Nutrition Research Center, Taipei Medical University Hospital, Taipei, Taiwan

**Keywords:** Dietary patterns, Body mass index, Underweight, Overweight, Obesity

## Abstract

**Background:**

Diet has been associated with differences in weight and nutritional status of an individual. The prevalence of overweight and obesity increased among adults in Taiwan. Hence, we examined the relationship between dietary patterns and weight status by gender among middle-aged and older adults in Taiwan.

**Methods:**

The cross-sectional data of 62,965 participants aged ≥40 years were retrieved from the Mei Jau health screening institutions’ database collected from 2001 and 2010. Diet information was evaluated using a food frequency questionnaire, while the dietary patterns were derived using principal component analysis before summing up and dividing into quintiles of consumption. The association between dietary patterns and weight status among adult men and women was explored using multinomial logistic regression models. Three models were analyzed before stratifying data by gender.

**Results:**

Two dietary patterns were derived with one reflecting a high consumption of vegetables and fruits (vegetable-fruit dietary pattern) and the other a high consumption of meat and processed foods (meat-processed dietary pattern). After adjustment, highest consumption of vegetables and fruits (Q5) reduced the likelihood of being overweight (OR = 0.91; 95% CI, 0.85–0.97) or obese (OR = 0.85; 95% CI, 0.78–0.92), while highest consumption of meat and processed foods increased the likelihood of being overweight (OR = 1.50; 95% CI, 1.40–1.59) or obese (OR = 1.94; 95% CI, 1.79–2.10). Women were less likely to be overweight or obese with the highest intake of fruits and vegetables (Q5) while both genders were more likely to be overweight or obese with high consumption of meat and processed foods.

**Conclusions:**

High intake of vegetables and fruits is associated with lower odds of being overweight or obese, especially among women. But, high intake of meat and processed foods is associated with higher odds of overweight and obesity in both genders.

**Electronic supplementary material:**

The online version of this article (10.1186/s12877-017-0664-4) contains supplementary material, which is available to authorized users.

## Background

In the past two decades, the prevalence of overweight and obesity in Taiwan among adults has increased from 33% to 44%, with the highest increase found among men (from 33% to 51%) than among women (from 33% to 36%) [1]. Being overweight or obese increases the risk of non-communicable diseases (NCDs) and some cancers such as endometrial, breast, and colon cancers [2]. Several factors such as dietary behavior, psychological factors, hereditary factors, and environmental factors have been linked to overweight and obesity, with diet being the most predominant factor leading to the imbalance between caloric intake and output [3]. Increases in the Western-style foods, refined foods, and high calorie foods, and a decrease in physical activity have been associated with weight changes [4, 5], and in recent years, dietary pattern in Taiwan has been characterized by a mixture of Chinese traditional diet and Western diet with an increased proportion of animal-based diet due to increased Western-style fast foods [6, 7]. To reduce the prevalence of obesity in Taiwan, it is important to consider changes in dietary behavior from a public health perspective and to promote the population wellbeing in general.

Authors using comprehensive analyses and factor analysis to derive dietary patterns in the Western population have also shown that plant-based dietary pattern and Western-style dietary pattern were negatively and positively associated with weight status, respectively [8]. Studies from Asian countries have also evidenced that diet is associated with weight changes [9–16], with most of these studies indicating a positive relationship between diet rich in meat and obesity [12–14, 16]. However, there is still conflicting evidence, in regard to vegetable-rich diet. Some authors found that a diet rich in vegetables lowers the risk of obesity [9], while others found that vegetable-rich diet is positively associated with obesity [11, 17]; and yet, others found a no association [13, 16]. These studies however were performed in different Asian countries. The previous cross-sectional study in Taiwan revealed that the vegetarians aged from 20 to 98 years had a lower risk of obesity compared with the matched non-vegetarians [18].

The increase in overweight and obese population in Taiwan may pose another challenge of NCDs, even if health promotion effort has been progressing in Taiwan, which may lead to more pressure on the current universal health care coverage system in Taiwan. Moreover, differences in diet cultures, food preparation methods, dietary environment, and anthropometric standards between the Eastern and Western countries add to the importance of a population-specific study on dietary patterns. Additionally, no studies have been reported on the association between dietary patterns and weight status by gender particularly among mid-aged and older adults in Taiwan. Therefore, the study investigated the association between dietary patterns and weight status among middle-aged and older adults in Taiwan. Furthermore, in order to extend the finding on obesity among Taiwanese adults [1], we explored whether the correlation between dietary patterns and weight status differed by gender.

## Methods

### Data source and subjects

The cross-sectional data of participants screened from 2001 to 2010 was acquired from a health screening institutions called Mei Jau (MJ) in Taiwan. The institutions collected detailed information on diet and weight status of adults at the point of entry during different time points across 10 year period. But, only data of all participants collected at the point of entry were used in our study. Following the rapid change in the weight status among adults in Taiwan [1], we only focused on adults who were 40 years and above from the dataset to explore our objectives. After eliminating those who had multiple entries, diabetes mellitus, renal disease, liver disorders, or any types of cancer, those taking lipid-lowering medications and had psychiatric disorder, and missing data in any of the variables used in this study, a total of 62,965 subjects (men: 32,735, 52%; women: 30,230, 48%) were used for our final analyses. The subjects with these diseases had usually been on the prescribed diet due to their health conditions. Data that had multiple entries of the subjects were excluded to avoid repeated measures from the same individual, while the first entry of the subjects with multiple entries was included consistently. If data collected over time from the same individual would have been included, it would have led to diagnostic suspicion bias and a reduction of a statistical power. We, therefore, used the baseline data (i.e. at the point of entry) of the participants in this study. However, it would be necessary for another study to assess the effect of a screening program on dietary changes and health outcome of the same participants.

### Ethical considerations

Taipei Medical University-Joint Institutional Review Board (TMU-JIRB) approved the study. All the subjects signed a written informed consent that the data would be used for academic research purposes only without their personal identification information. Participation was voluntary.

### BMI and weight status

Precision electronic weight scales were used to measure the body weight of the participants by the clinicians, while aluminum anthropometers were used for height measurements. Body weight and height of the participants were measured to calculate body mass index (BMI) as weight in kilograms divided by the square of height in meters. Weight status was defined using the Taiwan’s Ministry of Health and Welfare criteria where underweight, normal weight, overweight, and obesity are defined as BMI < 18.5 kg/m^2^, 18.5 kg/m^2^ ≤ BMI < 24 kg/m^2^; 24 kg/m^2^ ≤ BMI < 27 kg/m^2^; and BMI ≥ 27 kg/m^2^, respectively [19].

### Dietary assessment and patterns

The MJ Health Management Services developed, validated, and standardized a self-administered food frequency questionnaire (FFQ) that was used to assess participants’ diet. During the process of validation and standardization, twenty two food groups were classified from eighty five closed-ended questions based on previous studies [20–22]. The servings and frequency (per day or per week) of dietary intake were evaluated using FFQ with twenty two food groups (indicated in Additional file 1: Table S1) according to the consumption of food items at mealtimes [23, 24] in the past one month before data collection. For example, the intake of vegetables and root crops was assessed using the number of bowls per day (i.e., a bowl equals 11 cm in diameter). The intake of fruits as well as rice and flour products was measured using servings per day, while the responses for other food items were measured using servings per week. Examples in FFQ were given on the definition of one serving of food.

Dietary patterns were then derived using principal component analysis (PCA). A factor loading ≥0.30 was considered as the cut-off value to identify dietary patterns [25]. The food scores from 1 to 5 defined as the lowest to the highest frequency of intake in each dietary pattern were summed up before dividing into quintiles using *xtile* command in STATA software (StataCorp LP, College Station, Texas, USA).

### Other covariates

The other covariates that were also collected using a questionnaire included demographic, lifestyle, and health characteristics. The demographic and lifestyle characteristics were: gender, age group (i.e., 40–44, 45–49, 50–54, 55–59, and ≥60), education level (i.e., < high school, high school, and > high school), marital status (i.e., married, widows/divorced, and never married), smoking status (0 = no; 1 = yes), drinking alcohol (0 = no; 1 = yes), and physical activity (0 = no; 1 = yes). The question regarding smoking was binary defined as yes if the participant smoked cigarettes irrespective of the frequency and no if otherwise, the same was also for drinking alcohol. Physical activity was also defined as yes if the respondent engaged in exercises for more than one hour in a week and no if otherwise. The three health characteristics were: whether the participant has cardiovascular disease (CVD) defined as any disease that involves the heart or blood vessels (0 = no; 1 = yes), systolic blood pressure (SBP) > 120 mmHg (0 = no; 1 = yes), and diastolic blood pressure (DBP) > 80 mmHg (0 = no; 1 = yes). Participants who had been previously diagnosed with these health conditions, such as CVD, might have changed their lifestyle, and so the need to adjust for CVD indicator.

### Statistical analysis

We performed all statistical analyses using STATA version 13.1 (StataCorp LP, College Station, Texas, USA). Frequencies of demographic, lifestyle, health characteristics, and dietary patterns were determined according to weight status and compared using a chi-square test. Multinomial logistic regression was employed to determine the relationship between dietary patterns and weight status. Dummy variables were created for each of the variables to compare with the reference group. The reference group for vegetable-fruit and meat-processed dietary patterns was quintile 1 (Q1) which is the lowest or no food consumption, while the normal weight (i.e., BMI between 18.5 and 23.9 kg/m^2^) was the reference group for the weight status. Different models were derived. Model 1 was unadjusted while Model 2 was adjusted for age, education level, marital status, smoking, drinking, and physical activity; and Model 3 was adjusted for all the variables in Model 2 plus CVD, SBP, and DBP. Participants with prior diagnosis of CVD, SBP, and DBP may have had dietary modification in an effort to control these health characteristics, and hence controlling for the same was necessary. We finally stratified the analyses by gender to explore if gender is an effect modifier using the elaboration model. The *p*-value for trend of the dietary patterns was determined using the quintiles levels as a continuous indication. The alpha level of significance was *p* < 0.05.

## Results

### Dietary patterns

Two uncorrelated dietary patterns were derived from the FFQ using PCA with orthogonal varimax rotation and retention of two factors [26, 27], and defined as vegetable-fruit (9 foods or food groups) and meat-processed (12 foods or food groups) dietary patterns to reflect dietary consumption with high intake in vegetables and fruits or meats and processed foods, respectively (Additional file 1: Table S1). The food groups of legumes and soy products as well as seafood had a factor loading ≥0.30 in both factors, and were classified as vegetable-fruit or meat-processed dietary pattern, respectively.

Vegetable-fruit dietary pattern was characterized by high consumption of fruits, light-colored vegetables, dark-colored vegetables, root crops, legumes/soy products, vegetables with added oil/fat, milk, dairy products, and whole grains, while meat-processed dietary pattern was characterized by high consumption of meats, deep-fried foods, preserved and processed foods, organ meats, seafood, soy sauce or other dips, fried (rice and flour) products, sugary drinks, instant noodles, eggs, bread, and jam/honey. Only the food group of rice and flour products had a factor loading <0.30 in both factors, and was considered in neither vegetable-fruit nor meat-processed dietary pattern. Vegetable-fruit and meat-processed dietary patterns had a percentage of cumulative variance of 96.8% (46.4% and 50.4%, respectively) and eigenvalues greater than 1.0. These two dietary patterns were comparable to the Western-like dietary patterns but varied from approaches that include nutrients [26, 28, 29]. The theoretical maximum scores if a person consumed the entire vegetable-fruit dietary pattern (i.e., 9 food groups) and meat-processed dietary pattern (i.e., 12 food groups) were 45 and 60, respectively. However, the total score for vegetable-fruit dietary pattern was 42 (range, 9–42) while for meat-processed dietary pattern was 56 (range, 11–56). Table 1 shows the range of each quintile.

**Table 1:** Characteristics of the participants aged ≥ 40 years by weight status in Taiwan

		Weight status, %				
	All, %	Underweight	Normal weight	Overweight	Obese	*p* ^a^
*n*	62,965	2073	32,907	18,820	9165	–
Vegetable-fruit pattern						<0.001
Q1 (range, 9–14)	21.9	24.4	20.8	22.1	25.0	
Q2 (range, 15–16)	20.9	20.7	20.8	20.8	21.3	
Q3 (range, 17–18)	21.0	20.8	20.8	21.6	19.1	
Q4 (range, 19–21)	21.0	19.0	21.9	20.6	19.1	
Q5 (range, 22–42)	15.2	15.1	15.7	14.9	14.1	
Meat-processed pattern						<0.001
Q1 (range, 11–17)	21.3	24.8	22.3	19.9	19.8	
Q2 (range, 18–19)	18.4	19.3	19.5	17.5	16.8	
Q3 (range, 20–22)	26.6	27.0	26.8	26.6	25.7	
Q4 (range, 23–25)	18.5	16.2	18.0	19.4	18.9	
Q5 (range, 26–56)	15.1	12.9	13.4	16.5	18.8	
Gender, % male	52.0	34.7	45.7	62.7	56.4	<0.001
Age, years						<0.001
40–44	28.8	40.5	32.0	24.4	23.0	
45–49	20.9	19.3	21.8	20.6	18.6	
50–54	15.8	10.2	15.0	16.9	17.9	
55–59	11.4	7.6	10.3	12.7	13.7	
≥ 60	23.1	22.4	20.9	25.4	26.8	
Education level						<0.001
< High School	26.3	19.5	22.3	28.6	37.4	
High school	31.9	32.0	33.2	31.1	28.8	
> High school	41.8	48.5	44.5	40.3	33.7	
Marital status						<0.001
Married	86.3	80.7	85.9	87.8	85.8	
Widows/divorced	10.9	11.9	10.8	10.5	11.9	
Never married	2.8	7.4	3.4	1.7	2.3	
Smoking, current	19.6	19.1	17.8	22.1	20.8	<0.001
Drinking, current	18.6	11.7	16.6	22.2	20.4	<0.001
Physical activity, yes	63.9	55.9	64.6	65.8	59.7	<0.001
CVD	4.3	4.3	3.5	4.8	6.2	<0.001
SBP, > 120 mmHg	50.4	30.1	41.9	58.0	69.5	<0.001
DBP, > 80 mmHg	26.1	9.4	19.5	31.7	42.0	<0.001

^a^χ^2^ test for difference. *CVD* cardiovascular disease, *SBP* systolic blood pressure, *DBP* diastolic blood pressure

### Characteristics of participants

The characteristics of respondents were stratified across different weight status, and it showed that less than 20% of all the participants were in the highest quintile (Q5) of vegetable-fruit (15.2%) or meat-processed dietary pattern (15.1%) (Table 1). More than half of the respondents in the overweight group (62.7%) and obese group (56.4%) were males. In overall, majority of the total participants had high school education level or above (73.7%), were married (86.3%), were non-smokers (80.4%), were non-drinkers (81.4%), and had non-sedentary lifestyle (63.9%). On the other hand, only 4.3% of the respondents had been previously diagnosed with CVD, while 50.4% and 26.1% of the respondents had high SBP (>120 mmHg) and high DBP (> 80 mmHg), respectively. More than a half of the respondents in the overweight group (58.0%) and obese group (69.5%) had high SBP (> 120 mmHg). The chi-square test indicated a statistical difference in all the variables for the characteristics of the subjects at *p* < 0.001.

### Association between dietary patterns and weight status

The crude odds ratios of dietary patterns and other factors associated with weight status among middle-aged adults in Taiwan are shown in Table 2. The results showed those who had more intake of vegetable-fruit diet (Q2-Q5) were less likely to be underweight, overweight, or obese with exception of Q2 that was not statistically significant in the overweight group. However, those who consumed higher quantity of meat and processed foods (Q2-Q5) were more likely to be overweight or obese. In addition, men were more likely to be overweight (OR = 2.0, 95% CI = 1.93–2.07) or obese (OR = 1.53, 95% CI = 1.46–1.70) than women, and the odds ratio of being overweight or obese also increased with age. Moreover, the participants who had higher education level were less likely to be overweight or obese than those who had not attained high school graduation. Individuals with a higher education were more likely to be underweight (OR = 1.25; 95% CI, 1.11–1.41) and less likely to be overweight (OR = 0.71; 95% CI, 0.67–0.74) or obese (OR = 0.45; 95% CI, 0.43–0.48). Those who were smoking, drinking alcohol, had CVD, SBP > 120 mmHg, or DBP > 80 mmHg were also more likely to be overweight or obese. Physically active participants were not only less likely to be obese (OR = 0.81, 95% CI = 0.78–0.85) or underweight (OR = 0.70; 95% CI, 0.64–0.76), but were also more likely to be overweight (OR = 1.05; 95% CI, 1.02–1.09).

**Table 2:** Crude odds ratios of dietary patterns and characteristics by weight status of the participants aged ≥40 years in Taiwan, *n* = 62,965

	Model 1, Odds ratio (95% confidence interval)		
	Underweight *n* = 2073	Overweight *n* = 18,820	Obese *n* = 9165
Vegetable-fruit pattern			
Q1	1	1	1
Q2	0.85 (0.74, 0.97)*	0.94 (0.89, 0.99)*	0.85 (0.80, 0.99)***
Q3	0.86 (0.75, 0.98)*	0.97 (0.92, 1.03)	0.82 (0.76, 0.88)***
Q4	0.74 (0.65, 0.85)***	0.89 (0.84, 0.94)***	0.73 (0.68, 0.78)***
Q5	0.82 (0.71, 0.95)**	0.89 (0.84, 0.95)***	0.75 (0.69, 0.81)***
*P*-trend	< 0.001	< 0.001	< 0.001
Meat-processed pattern			
Q1	1	1	1
Q2	0.89 (0.78, 1.02)	0.99 (0.94, 1.06)	0.97 (0.90, 1.05)
Q3	0.90 (0.80, 1.02)	1.11 (1.05, 1.16)***	1.08 (1.00, 1.15)*
Q4	0.81 (0.70, 0.93)**	1.20 (1.13, 1.27)***	1.18 (1.09, 1.27)***
Q5	0.87 (0.74, 1.01)	1.38 (1.30, 1.47)***	1.57 (1.46, 1.70)***
*P*-trend	0.012	< 0.001	< 0.001
Gender			
Female	1	1	1
Male	0.63 (0.58, 0.69)***	2.00 (1.93, 2.07)***	1.53 (1.46, 1.61)***
Age, years			
40–44	1	1	1
45–49	0.70 (0.62, 0.78)***	1.24 (1.18, 1.31)***	1.19 (1.11, 1.28)***
50–54	0.53 (0.46, 0.63)***	1.48 (1.40, 1.57)***	1.67 (1.55, 1.79)***
55–59	0.58 (0.49, 0.69)***	1.61 (1.51, 1.72)***	1.85 (1.71, 2.01)***
≥ 60	0.84 (0.75, 0.95)**	1.59 (1.51, 1.68)***	1.78 (1.67, 1.91)***
Education level			
< High School	1	1	1
High school	1.10 (0.97, 1.25)	0.73 (0.70, 0.77)***	0.52 (0.49, 0.55)***
> High school	1.25 (1.11, 1.41)***	0.71 (0.67, 0.74)***	0.45 (0.43, 0.48)***
Marital status			
Married	1	1	1
Widows/divorced	1.17 (1.02, 1.35)*	0.95 (0.90, 1.01)	1.11 (1.03, 1.19)**
Never married	2.35 (1.97, 2.80)***	0.51 (0.45, 0.57)***	0.67 (0.58, 0.78)***
Smoking, current			
No	1	1	1
Yes	1.08 (0.97, 1.22)	1.31 (1.25, 1.37)***	1.21 (1.14, 1.28)***
Drinking, current			
No	1	1	1
Yes	0.67 (0.58, 0.76)***	1.43 (1.37, 1.50)***	1.29 (1.22, 1.37)***
Physical activity			
No	1	1	1
Yes	0.70 (0.64, 0.76)***	1.05 (1.02, 1.09)**	0.81 (0.78, 0.85)***
CVD			
No	1	1	1
Yes	1.24 (1.00, 1.55)	1.42 (1.30, 1.55)***	1.84 (1.66, 2.05)***
SBP, > 120 mmHg			
No	1	1	1
Yes	0.60 (0.54, 0.66)***	1.92 (1.85, 1.99)***	3.16 (3.00, 3.32)***
DBP, > 80 mmHg			
No	1	1	1
Yes	0.43 (0.37, 0.50)***	1.92 (1.84, 2.00)***	2.99 (2.84, 3.15)***

Normal weight is the reference group (*n* = 32,907). *CVD* cardiovascular disease, *SBP* systolic blood pressure, *DBP* diastolic blood pressure. Values with the star sign are significantly different from the reference group (indicated with an odds ratio of 1). * *p* < 0.05; ** *p* < 0.01; *** *p* < 0.001

After adjusting for demographic and lifestyle characteristics (Model 2) in Table 3, adults who were consuming more vegetables and fruits (Q5) were less likely to be overweight (OR = 0.91; 95% CI, 0.85–0.97) or obese (OR = 0.85; 95% CI, 0.78–0.92) while adults who were consuming more meat (Q5) were more likely to be overweight (OR = 1.50; 95% CI, 1.40–1.59) or obese (OR = 1.94; 95% CI, 1.79–2.10). Model 3 remained statistically significant with adjustment of health characteristics. Adults who had the highest consumption of vegetables-fruit dietary pattern (Q5) were still less likely to be overweight (OR = 0.92; 95% CI, 0.86–0.98) or obese (OR = 0.87; 95% CI, 0.80–0.94), while the ORs of the highest intake of meat and processed foods (Q5) indicated the likelihood of being overweight (OR = 1.48; 95% CI, 1.39–1.58) or obese (OR = 1.92; 95% CI, 1.77–2.08). There is somewhat a dose-response relationship, that is, with the increase in the quintile of consumption in the meat and processed foods pattern, the likelihood of being overweight and obese increased, while the vegetables-fruit dietary pattern exhibited a negative association with the increase in quintiles of consumption.

**Table 3:** Adjusted odds ratios (95% confidence interval) of the multinomial logistic regression of weight status among the participants aged ≥40 years in Taiwan, *n* = 62,965

	Men and women, Odds ratio (95% confidence interval)		
	Underweight *n* = 2073	Overweight *n* = 18,820	Obese *n* = 9165
Model 2			
Vegetable-fruit pattern			
Q1	1	1	1
Q2	0.89 (0.78, 1.02)	0.96 (0.91, 1.02)	0.92 (0.85, 0.98)*
Q3	0.92 (0.80, 1.05)	1.00 (0.95, 1.06)	0.91 (0.85, 0.98)**
Q4	0.80 (0.70, 0.92)**	0.92 (0.87, 0.97)**	0.82 (0.77, 0.89)***
Q5	0.92 (0.79, 1.07)	0.91 (0.85, 0.97)**	0.85 (0.78, 0.92)***
*P-*trend	0.059	< 0.001	< 0.001
Meat-processed pattern			
Q1	1	1	1
Q2	0.88 (0.77, 1.01)	1.04 (0.98, 1.10)	1.08 (1.00, 1.16)
Q3	0.88 (0.77, 1.00)*	1.18 (1.11, 1.24)***	1.25 (1.16, 1.34)***
Q4	0.78 (0.67, 0.90)***	1.29 (1.22, 1.37)***	1.42 (1.31, 1.53)***
Q5	0.81 (0.69, 0.95)**	1.50 (1.40, 1.59)***	1.94 (1.79, 2.10)***
*P*-trend	< 0.001	< 0.001	< 0.001
Model 3			
Vegetable-fruit pattern			
Q1	1	1	1
Q2	0.89 (0.78, 1.02)	0.97 (0.92, 1.03)	0.93 (0.86, 1.00)*
Q3	0.91 (0.80, 1.05)	1.01 (0.95, 1.07)	0.92 (0.85, 0.99)*
Q4	0.80 (0.69, 0.92)**	0.93 (0.88, 0.98)*	0.84 (0.78, 0.91)***
Q5	0.92 (0.79, 1.07)	0.92 (0.86, 0.98)**	0.87 (0.80, 0.94)***
*P*-trend	0.050	0.003	< 0.001
Meat-processed pattern			
Q1	1	1	1
Q2	0.88 (0.77, 1.01)	1.04 (0.98, 1.11)	1.08 (1.00, 1.17)*
Q3	0.88 (0.77, 1.00)*	1.18 (1.12, 1.24)***	1.25 (1.17, 1.35)***
Q4	0.78 (0.67, 0.90)***	1.29 (1.22, 1.37)***	1.41 (1.31, 1.53)***
Q5	0.82 (0.70, 0.96)*	1.48 (1.39, 1.58)***	1.92 (1.77, 2.08)***
*P*-trend	0.002	< 0.001	< 0.001

Normal weight is the reference group (*n* = 32,907); model 2 is adjusted for age, education level, marital status, smoking, drinking, and physical activity; model 3 is adjusted for model 2 plus cardiovascular disease, systolic blood pressure, and diastolic blood pressure. Values with the star sign are significantly different from the reference group (indicated with an odds ratio of 1).* *p* < 0.05, ** *p* < 0.01, *** *p* < 0.001

In the stratified analysis by gender in Table 4, the relationship between dietary patterns and weight status suggested that only women who consumed a diet rich in vegetables and fruits (Q5) were less likely to be overweight (OR = 0.86; 95% CI, 0.78–0.95) or obese (OR = 0.82; 95% CI, 0.72–0.93); though, Q4 and the trend *p*-value (*p* = 0.002) for men were statistically significant. On the other hand, those who consumed a diet high in meat and processed foods (Q3 to Q5) were more likely to be either overweight or obese both among men and women; also, the odds of being underweight were low in both men (Q2 to Q4, OR = 0.69–0.78) and women (Q4, OR = 0.81) who consumed meat and processed foods. In general, our results indicated a somewhat dose-response relationship between dietary intake and weight change.

**Table 4:** Multinomial logistic regression of weight status among the participants aged ≥40 years in Taiwan, stratified by gender, *n* = 62,965

	Stratified analysis by gender, Odds Ratio (95% confidence interval)^a^		
	Underweight *n* = 2073	Overweight *n* = 18,820	Obese *n* = 9165
Women, *n =* 30,230			
Vegetable-fruit pattern			
Q1	1	1	1
Q2	0.88 (0.74, 1.05)	0.92 (0.84, 1.01)	0.86 (0.77, 0.96)**
Q3	0.94 (0.89, 1.12)	0.99 (0.90, 0.07)	0.89 (0.79, 0.99)*
Q4	0.84 (0.71, 1.00)*	0.86 (0.79, 0.94)**	0.81 (0.72, 0.90)***
Q5	0.99 (0.81, 1.18)	0.86 (0.78, 0.95)**	0.82 (0.72, 0.93)***
*P*-trend	0.545	< 0.001	< 0.001
Meat-processed pattern			
Q1	1	1	1
Q2	0.97 (0.82, 1.15)	1.08 (0.99, 1.17)	1.10 (0.98, 1.22)
Q3	0.95 (0.82, 1.12)	1.17 (1.08, 1.27)***	1.36 (1.23, 1.50)***
Q4	0.81 (0.67, 0.97)*	1.28 (1.16, 1.40)***	1.34 (1.19, 1.51)***
Q5	0.82 (0.67, 1.02)	1.41 (1.27, 1.57)***	1.84 (1.62, 2.09)***
*P*-trend	0.016	< 0.001	< 0.001
Men, *n =* 32,735			
Vegetable-fruit pattern			
Q1	1	1	1
Q2	0.88 (0.71, 1.10)	1.00 (0.94, 1.09)	0.97 (0.88, 1.07)
Q3	0.87 (0.70, 1.09)	1.01 (0.94, 1.09)	0.92 (0.83, 1.01)
Q4	0.74 (0.58, 0.93)*	0.96 (0.89, 1.04)	0.85 (0.76, 0.93)***
Q5	0.76 (0.58, 0.99)*	0.97 (0.90, 1.06)	0.92 (0.81, 1.01)
*P*-trend	0.010	0.219	0.002
Meat-processed pattern			
Q1	1	1	1
Q2	0.69 (0.54, 0.89)**	1.02 (0.94, 1.11)	1.12 (1.00, 1.26)*
Q3	0.76 (0.61, 0.94)*	1.14 (1.06, 1.23)***	1.19 (1.07, 1.32)**
Q4	0.78 (0.62, 0.99)*	1.19 (1.09, 1.29)***	1.39 (1.24, 1.55)***
Q5	0.88 (0.68, 1.12)	1.34 (1.23, 1.46)***	1.80 (1.61, 2.01)***
*P*-trend	0.447	< 0.001	< 0.001

^a^Adjusted for age, education level, marital status, smoking, drinking, physical activity, cardiovascular disease, systolic blood pressure, and diastolic blood pressure. Normal weight is the reference group (*n* = 32,907). Values with the star sign are significantly different from the reference group (indicated with an odds ratio of 1).* *p* < 0.05, ** *p* < 0.01, *** *p* < 0.001

## Discussion

Our study examined the relationship between dietary patterns and weight status among middle-aged and elderly men and women in Taiwan. We found that men and women who consumed a diet high in meat and processed foods were more likely to be overweight or obese, unlike those who consumed a diet rich in vegetables and fruits. The prevalence of overweight and obesity from the year 1993 to 1996 increased in Taiwan from 33.4% to 51.0% in adult men and slightly in adult women from 33.5% to 35.9% [1]; and with these weight changes, in both men and women, dietary habits may have contributed to the increase in the prevalence of overweight and obesity. Previous studies also suggested that the change in lifestyle as well as in eating habit, from Chinese traditional dietary pattern to Western dietary pattern, could explain these relationships [30, 31]. According to the current methods of classifying dietary patterns in the Western countries, the vegetable-fruit dietary pattern could be referred to as ‘prudent and healthy’ dietary pattern [4].

In our study, the association between meat-processed foods dietary pattern and weight status did not attenuate among both men and women who were overweight or obese after adjusting for all the potential confounders. The evidence suggests that diet is a key factor of weight status and chronic disease like CVD [9, 32, 33]. Our finding also suggested that high intake of meat and processed foods by both men and women increased the chances of being overweight or obese.

In contrast to the previous studies which found that a diet high in vegetables was positively related with weight gain and obesity in China [11, 17], our results showed that adults who consumed a vegetable-rich diet were less likely to be overweight or obese. These differences may have been as a result of sedentary lifestyle, oily stir-fried vegetables, or even a change in dietary behavior in China, where those who are overweight or obese may have been advised to change eating habits, and were probably consuming a diet high in vegetables at the time the studies were conducted. More studies that would confirm this hypothesis are therefore necessary.

On the other hand, we found that high consumption of meat and processed foods increased the possibility of being overweight or obese. This supports the results of previous studies that diet rich in meat is associated with change in body weight among Asian adults of Chinese or Korean descent [12–14, 16]. The association between the meat-processed dietary pattern and weight status may be attributed to the mechanism of weight change with consumption of red meat and sugary foods as seen in this pattern. Studies have found that red meat is an energy-dense food containing saturated fat and cholesterol which may contribute to a surplus intake of energy, and hence increases the risk of overweight and obesity [34]. Additionally, high consumption of meat and processed foods may reveal some unnoticed dietary behavior or lifestyle which may have contributed to the weight differences as seen in this study. Typically, the traditional Chinese diet consists of plenty vegetables and low or moderate amounts of meat. But, with the increase in Western-style fast food outlet chains in Taiwan [6], it may have led to a change in dietary behavior from a more traditional Chinese diet to a Western-style diet that has more meat intake and less vegetable. Studies are therefore necessary to confirm this hypothesis. Even though it is frequently assumed that the primary reason of obesity is overeating combined with low physical activity, studies have shown that components of a diet may promote obesity even without excess energy consumption [35–37]. In general, our study highlighted the significance of meat and processed foods in increasing the prevalence of overweight and obesity among Taiwanese adults.

Our results also found that there was gender differences in weight status with women who consumed more vegetable and fruits (Q5) being less likely to be overweight or obese, and this was not the same for men at the same quintile level of consumption (Table 4). However, high consumption of meat and processed foods increased the likelihood of being overweight or obese in both genders.

### Strengths and limitations

Several strengths and limitations in the interpretation of our data must be considered. Some strengths included a large sample size and the use of a standardized validated questionnaire may have produced a consistent result of a more representative population in Taiwan. Secondly, to the best of our knowledge, our study is the first in Taiwan to examine the association between dietary patterns and weight status among a middle-aged and elderly population. Thirdly, the potential known confounders were controlled for, and this increased internal reliability. Finally, the criteria of defining weight status in Taiwan are different from the recommended World Health Organization–Asian criteria which define a normal weight range as 18.5–24.9 kg/m^2^, being overweight as ≥25 kg/m^2^, and being obese as ≥30 kg/m^2^ [38]. The difference in the cut-off criteria between Taiwan and Western countries shows that the problems and management of obesity may still be contentious, and hence more obesity-related studies with nationally derived criteria among different populations are still necessary. In this study, the nationally derived cut-off points are appropriate because of the difference in body structure between Asians and Caucasians. However, some of the limitations included the cross-sectional study design, of which, we are unable to establish causality between dietary patterns and weight status. A prospective cohort study would be more appropriate to explain and extend the finding of our study. Secondly, the multiple entries of the same individual were excluded in this study, and we could not monitor their dietary changes over time. Thirdly, the FFQ may not have been detailed enough to characterize the change in dietary behavior over time among Taiwanese, especially when the diet information of the past one month was collected as a snapshot and hence subjecting the data to a recall bias. Moreover, by using the food frequency data that were divided into quintiles of consumption, to indicate low and high consumption, it may also lead to misclassification bias where individuals who consuming vegetables and fruits more frequently are not certainly healthier compared with who with moderate frequency and vice versa. The recall bias also applies to self-reported measure of physical activity. Fourthly, we were unable to control for other important potential confounders, such as actual amount of energy and fat intake and household income among other potential confounders, due to data limitations. Finally, our subjects were confined to the middle-aged population in one health management institution in Taiwan, and hence our findings may not be generalized to the entire population of Taiwan. A population based study is necessary to extend the finding of our study. Therefore, our results must be interpreted with caution.

## Conclusions

In conclusion, our finding suggested that intake of a diet high in vegetables and fruits was strongly associated with lower odds ratios of overweight and obesity in women than in men, but a diet high in meat and processed foods was related with higher odds of overweight and obesity in both genders. However, the odds ratios were slightly higher in women consuming more meat and processed foods than those in men consuming the same diet. Future investigation could include longitudinal studies of obesity to determine the changes in dietary behavior over time and its effect on body weight in adulthood as well as the mechanism by which foods or active components of foods affect body weight. Despite the cross-sectional associations seen in our study, they may still have important for public health implications. Our findings support the current national Taiwan report indicating an increase in the prevalence of obesity. Public health promotion on changing dietary behavior is appropriate towards eating more vegetables, fruits, and whole grain and limiting high intake of meat and sugary foods in order to maintain healthy body weight in adulthood, mainly essential for men.

## Additional file

Additional file 1: Table S1. Factor loadings of 22 foods or food groups. (DOCX 16 kb)

## Abbreviations

BMI: body mass index
CVD: cardiovascular disease
DBP: diastolic blood pressure
FFQ: food frequency questionnaire
NCDs: non-communicable diseases
PCA: principal component analysis
SBP: systolic blood pressure
TMU-JIRB: Taipei Medical University-Joint Institutional Review Board
