# Supplementary material for: Dietary patterns, gender, and weight status among middle-aged and older adults in Taiwan: a cross-sectional study
Source: BMC Geriatr. 2017 Nov 21;17:268. doi: 10.1186/s12877-017-0664-4 (PMC5696781; doi:10.1186/s12877-017-0664-4)
Supplement: Additional file 1: Table S1. — Factor loadings of 22 foods or food groups. (DOCX 16 kb) [file 12877_2017_664_MOESM1_ESM.docx]

Table S1 Factor loadings of 22 foods or food groups

| Food or food group | Factor 1  (vegetable-fruit dietary pattern) | Factor 2  (meat-processed dietary pattern) |
| --- | --- | --- |
| Dairy products | 0.33* | 0.13 |
| Legumes and soy products | 0.38* | 0.31* |
| Light-colored vegetables | 0.76* | -0.06 |
| Dark-colored vegetables | 0.79* | -0.09 |
| Fruits | 0.57* | -0.06 |
| Whole grains | 0.33* | 0.09 |
| Root crops | 0.46* | 0.23 |
| Milk | 0.31* | -0.10 |
| Meats | 0.16 | 0.50* |
| Seafood | 0.33* | 0.33* |
| Organ meats | 0.06 | 0.51* |
| Bread | 0.23 | 0.32* |
| Sugary drinks | -0.07 | 0.46* |
| Preserved and processed foods | 0.01 | 0.59* |
| Eggs | 0.20 | 0.41* |
| Vegetables with added oil/fat | 0.52* | 0.15 |
| Rice and flour products | 0.25 | 0.21 |
| Fried rice and flour products | 0.13 | 0.47* |
| Jam/honey | 0.17 | 0.31* |
| Deep-fried foods | 0.04 | 0.62* |
| Instant noodles | -0.11 | 0.42* |
| Soy sauce or other dips | -0.02 | 0.54* |

*A factor loading ≥ 0.30 which is considered as the cut-off for the identification of dietary patterns.
